# Astragalus Polysaccharide Enhances O‐GlcNAcylation Through OGT to Improve Intervertebral Disc Degeneration in Rats

**DOI:** 10.1111/jcmm.70940

**Published:** 2025-12-10

**Authors:** Hao Tan, Cao Fang, Yiyun Tan, Zhi Wang, Yun Zhou, Xing Li

**Affiliations:** ^1^ Department of Orthopedics Changsha Hospital of Traditional Chinese Medicine (Changsha Eighth Hospital) Changsha China; ^2^ Orthopedic Rehabilitation Center Hunan Provincial Occupational Disease Prevention and Treatment Hospital (Occupational Disease Prevention and Treatment Hospital Affiliated to University of South China) Changsha China

**Keywords:** astragalus polysaccharides, intervertebral disc degeneration, Nrf2, O‐GlcNAcylation, OGT

## Abstract

Astragalus polysaccharides (APS) are a crucial bioactive component known for their various pharmacological properties. Abnormal O‐linked β‐N‐acetylglucosamine modification (O‐GlcNAcylation) is noted in cases of intervertebral disc degeneration (IVDD). Nonetheless, it remains uncertain whether APS regulates the process of O‐GlcNAcylation associated with IVDD. We employed molecular docking, cycloheximide chase assay, immunohistochemistry, and immunoprecipitation to investigate APS‐mediated OGT/O‐GlcNAcylation regulation of Nrf2. The effects of APS and its role in promoting the O‐GlcNAcylation of Nrf2 in IVDD through both in vivo and in vitro studies are discussed. In vitro investigations demonstrated an increase in the levels of OGT and O‐GlcNAcylation in nucleus pulposus cells (NPCs) following exposure to tert‐butyl hydroperoxide (TBHP). APS further facilitated improvements in OGT expression and O‐GlcNAcylation processes, restoring the viability of NPCs inhibited by TBHP and promoting the synthesis of collagen II and aggrecan, while reducing apoptosis. Mechanistically, APS promotes the expression of OGT by targeting it. Furthermore, O‐GlcNAcylation mediated by OGT stabilizes the expression of Nrf2 via the ubiquitin‐proteasome pathway. Rescue experiments indicated that the disruption of either OGT or Nrf2 expression negated the protective role of APS on NPCs. Ultimately, both in vitro and in vivo studies indicated that APS significantly enhanced OGT expression and O‐GlcNAcylation, which subsequently improved Nrf2 expression and contributed to the alleviation of IVDD in rats. APS promotes O‐GlcNAcylation through OGT, thereby stabilizing the expression of Nrf2, which in turn contributes to the improvement of IVDD.

## 1 Introduction

Despite significant advancements in surgical techniques and healthcare, over 80% of the global population experiences low back pain (LBP) at some stage in their lives, severely impacting life quality [1]. Intervertebral disc degeneration (IVDD) is recognised as the primary cause of LBP [2]. IVDD is linked to alterations in the functionality of the nucleus pulposus (NP), annulus fibrosus (AF), and cartilage endplate (EP) [3]. NP cells (NPCs), fundamental elements in NP tissues, are vital for the synthesis of the extracellular matrix (ECM) and the maintenance of NP environmental balance [4]. Recent research indicates that the progression of IVDD is linked to a variety of pathological factors, including oxidative stress, aging, ECM degradation, endoplasmic reticulum stress, and inflammatory responses [5, 6, 7]. However, current treatment options are largely limited to delaying the progression of the condition, with no effective strategies available to reverse the biological degeneration associated with IVDD. Therefore, further understanding of the pathological mechanisms underlying IVDD is vital.

Recently, natural products derived from plants and their by‐products have gained recognition as promising therapeutic options in clinical settings, attributed to their unique pharmacological properties [8, 9]. The species Astragalus membranaceus (Fisch.) Bge, a traditional medicinal herb from China, demonstrates remarkable therapeutic potential in the treatment of various clinical conditions [10]. Its bioactive constituents include cycloastragenol, Astragalus saponin IV (AS‐IV), and Astragalus polysaccharides (APS), among others [11]. Cycloastragenol and AS‐IV have been shown to activate telomerase, thereby protecting NPCs from aging and apoptosis induced by elevated glucose levels [12]. Research indicates that AS‐IV offers protective benefits against IVDD in murine models [13]. Nonetheless, the primary active component, APS, has not been extensively studied regarding its regulatory effects on NPCs and its potential therapeutic implications for IVDD.

O‐linked β‐N‐acetylglucosamine modification (O‐GlcNAcylation) represents a significant, dynamic, and reversible form of post‐translational modification [14]. This modification plays a crucial role in maintaining optimal homeostasis through the reciprocal regulation of O‐GlcNAc transferase (OGT) and O‐GlcNAcase (OGA) [15]. Recent studies have established a correlation between the dysregulation of O‐GlcNAcylation and the pathogenesis of multiple human diseases, including cancer, osteoarthritis, and neurodegenerative disorders [16, 17, 18]. Notably, an enhanced expression of O‐GlcNAcylation and OGT has been observed in degenerated disc tissue in humans [19]. However, the specific roles and underlying mechanisms of O‐GlcNAcylation and OGT in IVDD remain largely unexplored.

Emerging evidence suggests that the activation of the nuclear factor E2‐related factor 2 (Nrf2) signalling pathway is essential for mitigating the progression of IVDD [20, 21]. The stimulation of Nrf2 signalling has been shown to protect NPCs from oxidative stress and apoptosis [22]. Moreover, previous research conducted by Han et al. has demonstrated the capacity of APS to activate the Nrf2 signalling pathway [23]. Nevertheless, the potential modification of Nrf2 through O‐GlcNAcylation induced by APS has not yet been studied. Therefore, the present study seeks to investigate the therapeutic role of APS in IVDD progression and to elucidate the regulatory mechanism underlying the O‐GlcNAcylation modification of Nrf2.

## 2 Materials and Methods

### 2.1 Establishment of IVDD Model

Eight‐week‐old male Sprague–Dawley (SD) rats (Slake Jingda, China) were used to develop the IVDD model. The study protocols were approved by Changsha Hospital of Traditional Chinese Medicine Animal Ethics Committee (No. 2023072011). Following a one‐week acclimatisation period, the IVDD model was set up through the pointed injury technique, as previously mentioned [24]. Specifically, following the anaesthesia of the rats with an intraperitoneal dose of pentobarbital (40 mg/kg), the Caudal Vertebra 7/8 (Co7/8) measurement was ascertained via manual palpation. A 22‐G sterile needle was employed to puncture the central annulus fibrosus layer of the Co7/8 vertically to a depth of 4 mm, followed by a 360° rotation for 15 s. The rats in the Sham group did not receive any intervention.

In the investigation of the treatment for IVDD in rats with APS, subjects were administered varying daily doses of APS (200, 500, 800 mg/kg/day, Aladdin, 708487, China) commencing the day following surgical intervention [25]. The other groups received equivalent volumes of normal saline. The rats were permitted unrestricted movement and were closely monitored to ensure their welfare.

To study the effects of O‐GlcNAcylation on the IVDD rats, OGT small hairpin RNA (sh‐OGT; 5 nmol, 10 μL) and a control plasmid (sh‐NC) were administered via injection into the L3/4 intervertebral disc [26]. All rats were euthanized eight weeks post‐surgery, and the corresponding disc tissues were collected for subsequent analysis.

### 2.2 Histological Staining

Following fixation with 4% paraformaldehyde, the rat intervertebral disc was decalcified using a decalcification solution. The fixed intervertebral disc was encapsulated in paraffin wax and cut into 6 μm thick sections. Prior to haematoxylin and eosin (H&E) and saffron O‐fast green staining, the paraffin sections were baked at 60°C for 2–3 h and then treated with xylene for 20 min, three times, followed by sequential immersions in 100%, 95%, 85%, and 75% ethanol for 5 min each, and finally soaked in distilled water for 5 min. Following the de‐waxing, sections were stained according to the H&E (AWI0001a, AWI0029a, Abiowell) and saffron O‐fast green (AWI0240a, Abiowell) staining kit instructions, and pictures were taken using an optical microscope (BA210T, Motic, China).

For immunohistochemical (IHC) staining, after deparaffinizing and executing antigen repair, the paraffin sections were incubated with Collagen II (28459‐1‐AP, Proteintech, USA), aggrecan (13880‐1‐AP, Proteintech), or O‐GlcNAc (677902, BioLegend, USA) overnight. On the second day, they were incubated with HRP‐labelled secondary antibody for 30 min at 37°C. Following DAB staining, the nuclei were restained with haematoxylin. The slides were finally sealed using neutral gum, and images were captured under a light microscope.

### 2.3 Isolation and Culture of NPCs

NPCs were extracted from SD rats under sterile conditions [24, 27]. Briefly, 2–3‐week‐old SD rats were euthanized via cervical dislocation post‐anaesthesia. Six to eight caudal vertebrae were procured from the base of the tail using a surgical blade. The AF of the disc was cut to retrieve the normal rat NP tissue. The NP tissue was then dissected into 1 cubic millimetre sections. These sections were digested at 37°C for 1 to 2 h, with gentle agitation, in 4 mL of 0.2% type 2 collagenase and subsequently filtered through a 70‐μm filter to harvest the rat NPCs. NPCs were cultured in DMEM/F12 medium augmented with 20% FBS in a 5% CO_2_ incubator at 37°C. NPCs from the second or third generation were utilized for subsequent experiments.

### 2.4 Cell Intervention

To investigate the role of APS in vitro in IVDD, NPCs were treated with 30 μM TBHP (B106035, aladdin, China) for 24 h [24], followed by exposure to varying concentrations of APS (0.1, 0.2, 0.4, 0.8, and 1.6 mg/mL) for 24 h [25]. The effects of these treatments on cell viability, apoptosis, and ECM degradation of NPCs were then examined.

siRNA Nrf2 (si‐Nrf2), OGT knockdown plasmid (sh‐OGT), and control plasmid (HonorGene, China) were transfected into the cells, respectively. Transfection was carried out using Lipofectamine 2000 reagent (11668‐019, Invitrogen, USA). TBHP and APS were then added after 48 h.

### 2.5 Quantitative Reverse Transcription PCR (qRT‐PCR)

Total RNA from tissues and cells was isolated using TRIZOL reagent (15596026, ThermoFisher, USA). RNA was then reverse transcribed into cDNA using the HiFi‐Script cDNA first strand synthesis kit (CW2569, CWBio, China). Quantitative PCR was carried out using the UltraSYBR Mixture kit (CW2601, CWBio). Data were analysed using the 2^−ΔΔCt^ method, normalized to β‐actin (Table 1).

**TABLE 1:** Primers sequence of qRT‐PCR.

Name of primer	Sequences (5′‐3′)
Collagen II‐F	ACCCTCAACCCCAAAACAACACA
Collagen II‐R	TCAGGTCAGCCATTCAGTGC
Aggrecan‐F	ACAGACACCCCTACCCTTGC
Aggrecan‐R	CCTCACATTGCTCCTGGTCGAT
Nrf2‐F	ACGGCTAAAACTTCCTACTGTGA
Nrf2‐R	ACACTTACACAGAAACTAGCCCAA
β‐actin‐F	ACATCCGTAAAGACCTCTATGCC
β‐actin‐R	TACTCCTGCTTGCTGATCCA

### 2.6 Western Blot (WB)

Total proteins were extracted from tissues and cells using RIPA lysate (P0013B, Biyuntian, China). A BCA protein concentration assay kit (PC0020, Solarbio, China) was used to determine the concentration of the samples. The protein was isolated using 10% SDS‐polyacrylamide gel. The separated proteins are transferred to a nitrocellulose filter membrane, which is then blocked with 5% skim milk powder (AWB0004, Abiowell). The membranes were incubated with primary and secondary antibodies (Table 2). Finally, protein expression was detected using SuperECL Plus hypersensitive luminescent solution (K‐12045‐D50, advansta, USA). Furthermore, for immunoprecipitation (IP), after lysing the cells with IP lysis buffer, O‐GlcNAc antibody (65292‐1‐Ig, Proteintech) and Protein A/G magnetic beads were added for incubation. The expressions of O‐GlcNAc and Nrf2 were analyzed according to the above method.

**TABLE 2:** Antibody information.

Name	Cat. number	Company	Country
Bax	ab32503	Abcam	UK
Bcl2	ab182858	Abcam	UK
Cleaved Caspase‐3	#9664	CST	USA
Collagen II	AWA59143	Abiowell	China
Aggrecan	AF301310	AiFang	China
OGT	66823‐1‐Ig	Proteintech	USA
O‐GlcNAc	65292‐1‐Ig	Proteintech	USA
OGA	14711‐1‐AP	Proteintech	USA
Nrf2	#12721	CST	USA
HO‐1	10701‐1‐AP	Proteintech	USA
β‐actin	AWA80002	Abiowell	China
HRP goat anti‐mouse IgG	SA00001‐1	Proteintech	USA
HRP goat anti‐rabbit IgG	SA00001‐2	Proteintech	USA

### 2.7 Detection of Apoptosis

The evaluation of apoptotic cells in intervertebral discs was performed using a TUNEL assay kit (YEASEN, 40306ES50) In brief, following the de‐waxing of the paraffin section, it was cleaned twice with PBS. The slices were then incubated with a TUNEL reaction mixture of 50 μL in a humidified environment at 37°C for 1 h. The nucleus was then restained with DAPI. The resulting images were analysed with fluorescence microscopy (BA210T, Motic, China) and Image Pro Plus 6.0 software, which was used to determine the percentage of apoptotic cells in relation to the total cell count, effectively giving the apoptotic rate.

To detect the apoptosis of NPCs in each group, an Annexin V/PI double staining kit (KGA1030, KeyGen, China) was utilized. In brief, the cells were collected and re‐suspended in the binding buffer. They were then stained with 5 μL of annexin V‐fluorescein isothiocyanate (FITC) and 10 μL of propidium iodide (PI). Following a 10‐min incubation period at room temperature, early and late apoptotic NPCs were analyzed and counted using flow cytometry (A00‐1‐1102, Beckman, USA).

### 2.8 Cell Counting Kit‐8 (CCK‐8)

NPCs (5 × 10^3^ cells/well) were seeded into a 24‐well plate at a volume of 300 μL per well. The NPCs were exposed to APS, and 30 μL/well of the CCK‐8 solution (CK04, Dojindo, Japan) was added. The cells were then left to incubate at 37°C with 5% CO_2_ for a duration of 4 h. A microplate reader (MB530, HEALES, China) was utilized to determine the optical density (OD) values of each well at 450 nm.

### 2.9 Cycloheximide (CHX) Tracing Method

NPCs were pre‐incubated with 10 μM Thiamet G or OSMI‐4 or 0.8 mg/mLAPS for 24 h prior to being treated with 50 μM CHX for multiple periods (2, 4, 6, 8 h). The cells were then harvested at the corresponding times, and the extracted protein was analyzed to determine Nrf2 protein degradation via immunoblotting.

### 2.10 Ubiquitination Detection

Following pre‐incubation of NPCs with 10 μM Thiamet G or OSMI‐4 for 24 h, they were exposed to 10 μM MG132 for an additional 4 h. Following cell lysis with IP lysate, the lysate was treated with anti‐ubiquitin (10201‐2‐AP, Proteintech) or an anti‐IgG antibody. After incubating with the antibody overnight, the cell lysate was added to pre‐treated protein A/G agarose beads and incubated for another 2 h. After co‐immunoprecipitation, the level of Nrf2 ubiquitination was detected via immunoblotting.

### 2.11 Molecular Docking

Download the three‐dimensional structure diagram of APS from the PubChem database. Download the three‐dimensional structure of the OGT protein from the RCSB PDB database. Use the AutoDock Vina molecular docking software to conduct molecular docking analysis of the OGT protein and APS.

### 2.12 Data Statistics and Analysis

Statistical analysis was performed using GraphPad Prism 8.0 software. Quantitative data were represented as mean ± standard deviation. Unpaired *t*‐tests were used for comparisons between two groups, and one‐way ANOVA was applied for comparisons among multiple groups, followed by Tukey's post hoc test. A *p*‐value of less than 0.05 is considered statistically significant.

## 3 Results

### 3.1 APS Alleviates IVDD in Rats

**FIGURE 1:**
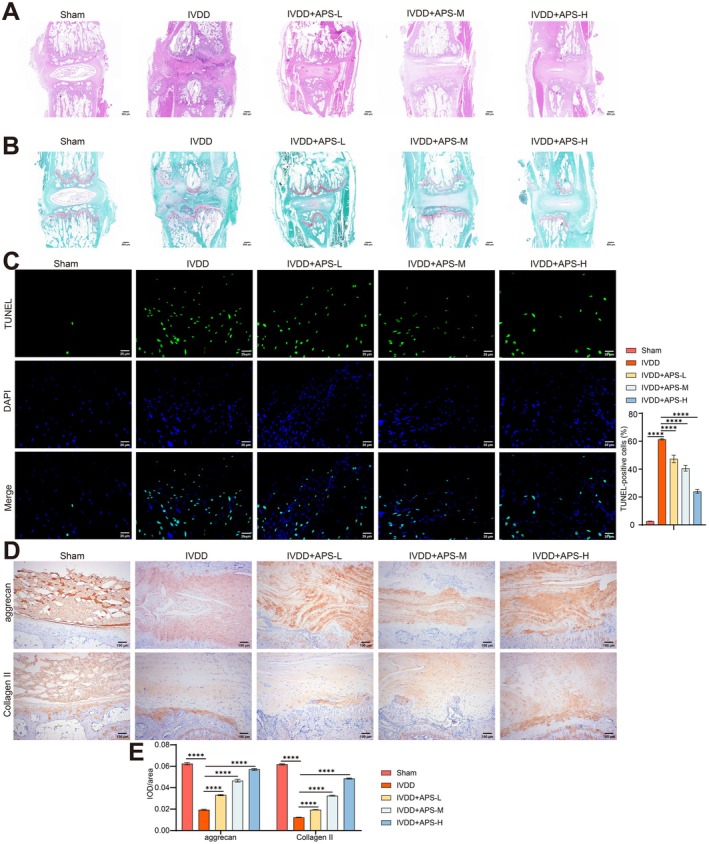
APS alleviates IVDD deterioration in rats. IVDD rats were treated with low (200 mg/kg/d), medium (500 mg/kg/d) and high (800 mg/kg/d) doses of APS to explore the role of APS in IVDD in vivo. (A–C) Representative images of rat intervertebral disc tissue stained with H&E, saffron O‐fast green, and TUNEL staining. (D, E) IHC images (D) and statistic analysis (E) of Collagen II and aggrecan. *n* = 5. ****p < 0.0001.

To investigate the potential therapeutic role of APS in IVDD, we established a rat IVDD model and orally administered APS via gavage. Histological examinations utilizing H&E and saffron O‐fast green staining indicated that the intervertebral discs in the sham group exhibited normal structural integrity. In contrast, the IVDD group displayed significant pathological alterations, characterized by notable NP atrophy and pronounced structural damage to AF. Treatment with APS resulted in a significant reduction of these pathological changes in the intervertebral discs of the IVDD rats. Notably, APS therapy at a dosage of 800 mg/kg/day (APS‐H group) showed superior outcomes compared to lower dosages of 500 mg/kg/day (APS‐M group) and 200 mg/kg/day (APS‐L group) (Figure 1A,B).

Additionally, TUNEL staining results showed a tremendously higher incidence of apoptosis in NP and AF samples from the IVDD group compared to the sham group (Figure 1C). According to IHC staining data, expression of Collagen II and aggrecan in the IVDD group disc samples exhibited a marked reduction (Figure 1D,E). Conversely, the introduction of APS appeared to counteract these detrimental changes compared to the IVDD group, with the APS‐H group showing the most effective intervention performance (Figure 1C–E). Therefore, a dosage of 800 mg/kg/day was chosen for subsequent APS treatment in IVDD rats. To sum up, these results validate the efficacy of APS in ameliorating IVDD in rats.

### 3.2 APS Ameliorates NPCs Apoptosis and ECM Degradation

**FIGURE 2:**
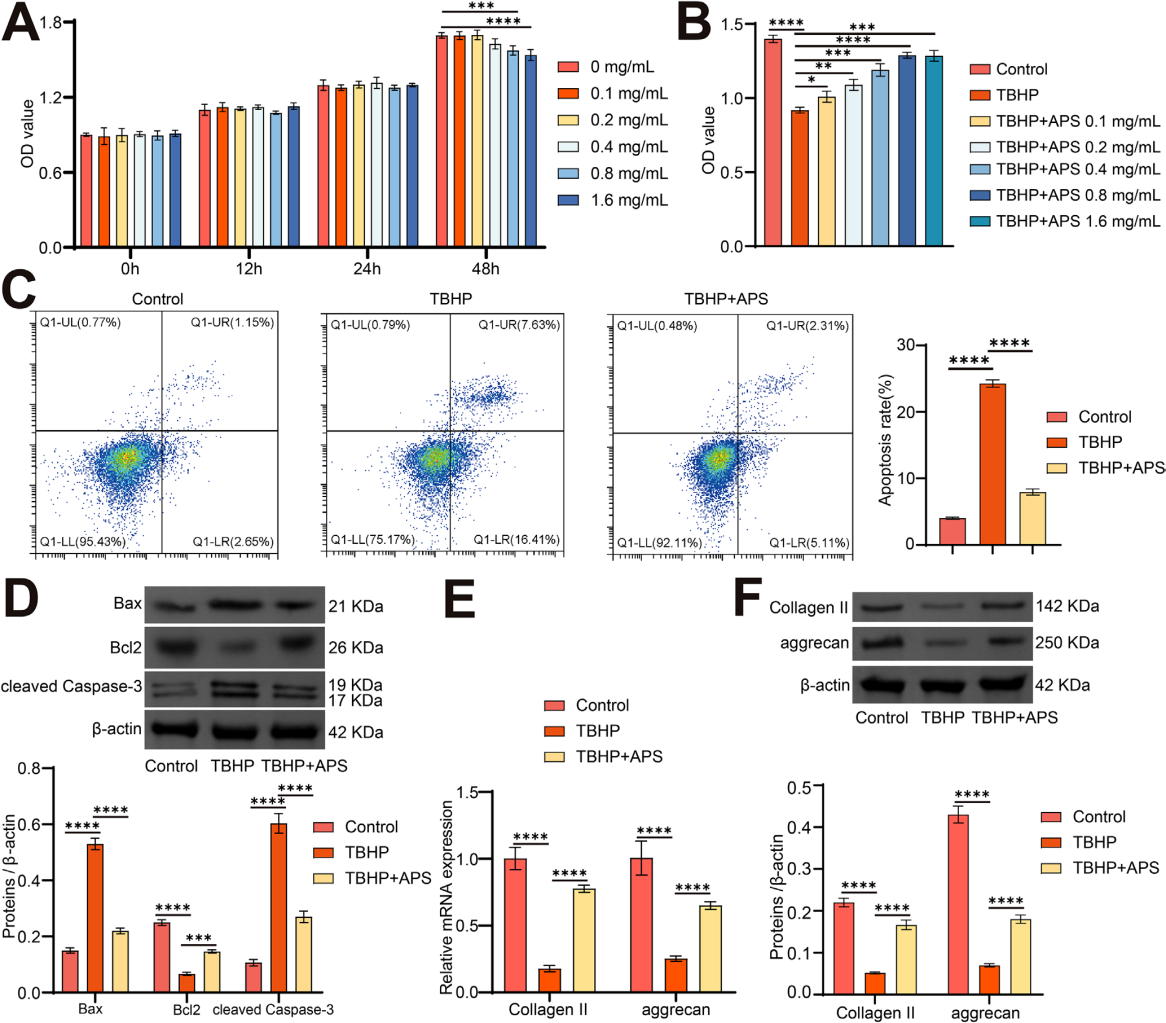
APS ameliorates NPCs apoptosis and ECM degradation. NPCs were stimulated with 30 μM TBHP for 24 h and then treated with APS at different concentrations (0.1, 0.2, 0.4, 0.8 and 1.6 mg/mL) for 24 h to explore the effects of APS on the function of NPCs in vitro. (A) The cytotoxicity of NPCs treated with varying concentrations of APS for 12, 24, and 48 h was evaluated using the CCK8 assay. (B) The CCK8 assay was used to assess the effects of varying concentrations of APS on cell viability in TBHP‐induced conditions. (C) Annexin V‐PI was utilised for apoptosis detection in NPCs. (D) The protein levels of Bcl‐2, Bax, and cleaved caspase 3 were assessed by WB. E‐F. The changes in the expression of Collagen II and aggrecan were examined via qRT‐PCR (E) and WB (F). *n* = 3. *p < 0.05, **p < 0.01, ***p < 0.001, ****p < 0.0001.

Research indicates that NPCs embedded within the ECM are instrumental in the regulation of IVDD [28, 29]. Consequently, NPCs were designated as the target cells for the subsequent investigation. To investigate the therapeutic potential of APS in an in vitro model of IVDD, we initially treated NPCs with a range of APS concentrations (0, 0.1, 0.2, 0.4, 0.8, and 1.6 mg/mL). No significant cytotoxic effects on the NPCs were observed across these concentrations according to the CCK8 assay, despite a decrease noted at 48 h (Figure 2A).

To replicate the pathological conditions associated with IVDD in vitro, NPCs were treated with TBHP. The CCK8 assay revealed that TBHP treatment substantially reduced the viability of NPCs compared to the control group. However, APS intervention improved the cell viability of NPCs in a dose‐dependent manner compared to the TBHP group, with APS at 1.6 mg/mL showing similar effects to the 0.8 mg/mL dosage (Figure 2B). As a result, we opted to use 0.8 mg/mL APS for subsequent cell experiments.

Flow cytometry demonstrated that the number of apoptotic NPCs notably increased following TBHP treatment compared to the control group (Figure 2C). WB analysis showed that TBHP treatment elevated the protein levels of Bax and cleaved caspase 3 while inhibiting the protein expression of Bcl2 compared to the control group (Figure 2D). Furthermore, alterations in key ECM degradation genes were detected by qRT‐PCR and WB, revealing that TBHP treatment had inhibited the expression of Collagen II and aggrecan compared to the control group (Figure 2E,F). However, APS intervention greatly attenuated these IVDD‐associated cellular activities (Figure 2C–F). These results suggest that APS intervention effectively inhibits NPCs' apoptosis and restores the balance of ECM degradation under IVDD conditions.

### 3.3 APS Enhances O‐GlcNAcylation Modification

**FIGURE 3:**
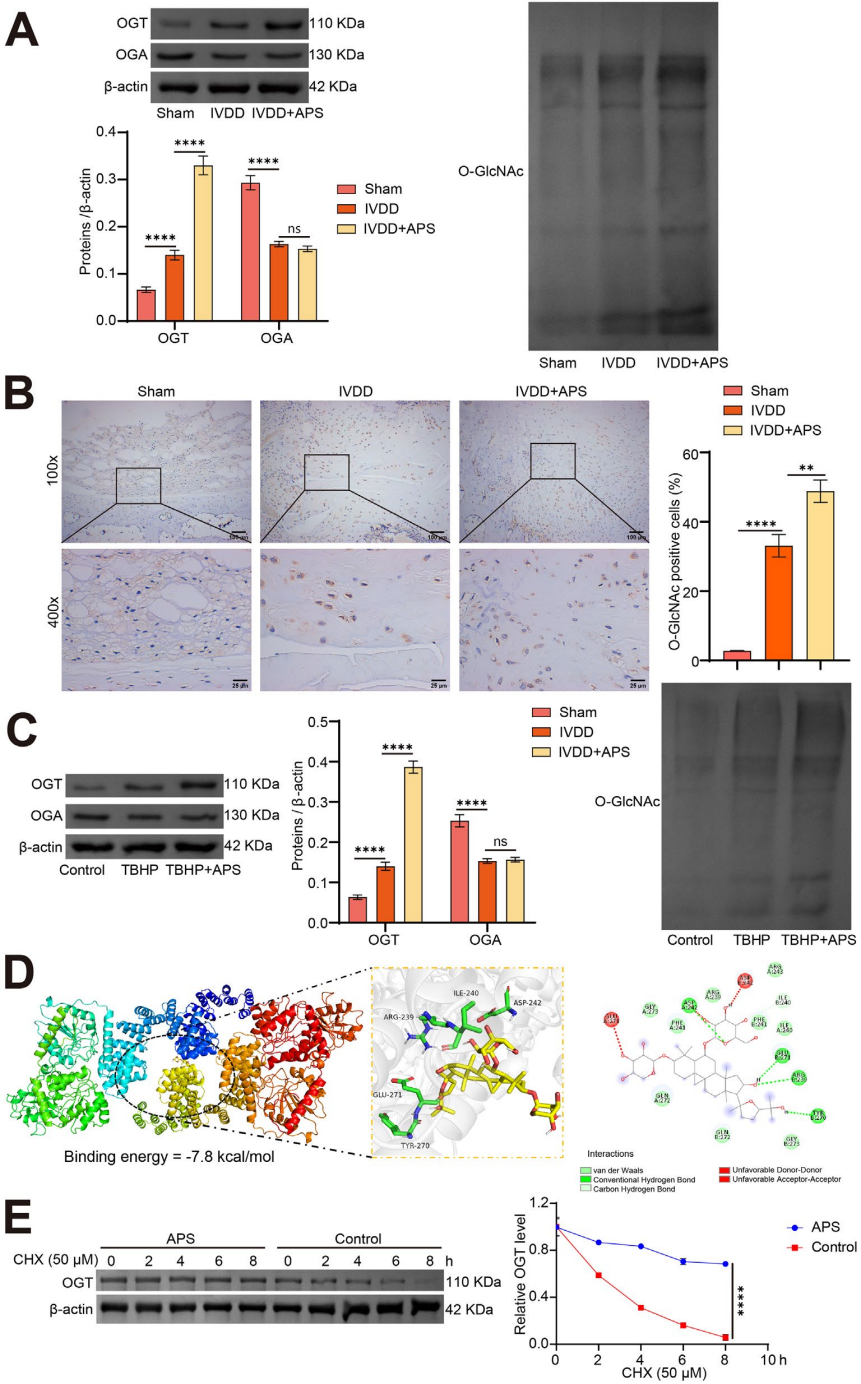
APS enhances O‐GlcNAcylation modification. IVDD rats were treated with 800 mg/kg/d APS to explore the regulatory effect of APS on O‐GlcNAcylation in IVDD. (A) WB was employed to determine the protein levels of OGT, OGA, and O‐GlcNAc in intervertebral disc tissue. (B) The positive rate of O‐GlcNAc in intervertebral disc tissues was detected by IHC. (C) The protein levels of OGT, OGA, and O‐GlcNAc in NPCs were evaluated through WB. (D) Molecular docking analysis of APS and OGT. (E) The effect of APS on the stability of OGT protein was detected by using CHX chase test. (A, B), *n* = 5. (C, E), *n* = 3. **p < 0.01, ****p < 0.0001, ns indicates *p* > 0.05.

Recent studies have underscored the regulatory role of APS on O‐GlcNAcylation [30]. Hence, this section of the study aimed to determine whether O‐GlcNAcylation is involved in the protective effects of APS against IVDD. WB results showed that compared to the Sham group, the protein levels of OGT and O‐GlcNAc tremendously increased in the intervertebral disc tissue from the IVDD group, while OGA levels notably decreased. APS treatment further augmented the expression of OGT and O‐GlcNAc but did not affect the expression of OGA (Figure 3A). IHC staining further confirmed these shifts in O‐GlcNAc expression within the intervertebral disc tissues (Figure 3B). Moreover, we observed that protein levels of OGT and O‐GlcNAc tremendously increased, while OGA levels decreased in the TBHP group compared to the Control group. Intriguingly, APS treatment further elevated levels of OGT and O‐GlcNAc proteins, but did not affect the expression of OGA (Figure 3C).

Further, we conducted molecular docking analysis between APS and OGT. The results showed that the binding energy of APS and OGT was −7.8 kcal/mol. As shown in Figure 3D, APS mainly interacts with the OGT protein through hydrogen bonds. APS can form stable hydrogen bonds with ASP 242 on the A chain of the OGT protein and with ILE 240, GLU 271, ARG 239, and TYR 270 on the B chain of the OGT protein. These interactions facilitate the binding of APS to the active site of the OGT protein (Figure 3D). The results of the CHX tracing experiment analyzing the stability changes of OGT showed that compared with the control group, the protein degradation rate of OGT was slowed down after APS treatment (Figure 3E). These findings suggest that APS enhances O‐GlcNAcylation by targeting OGT during the intervention of IVDD, both in vitro and in vivo.

### 3.4 O‐GlcNAcylation Stabilises Nrf2 Expression Through the Ubiquitin‐Proteasome Pathway

**FIGURE 4:**
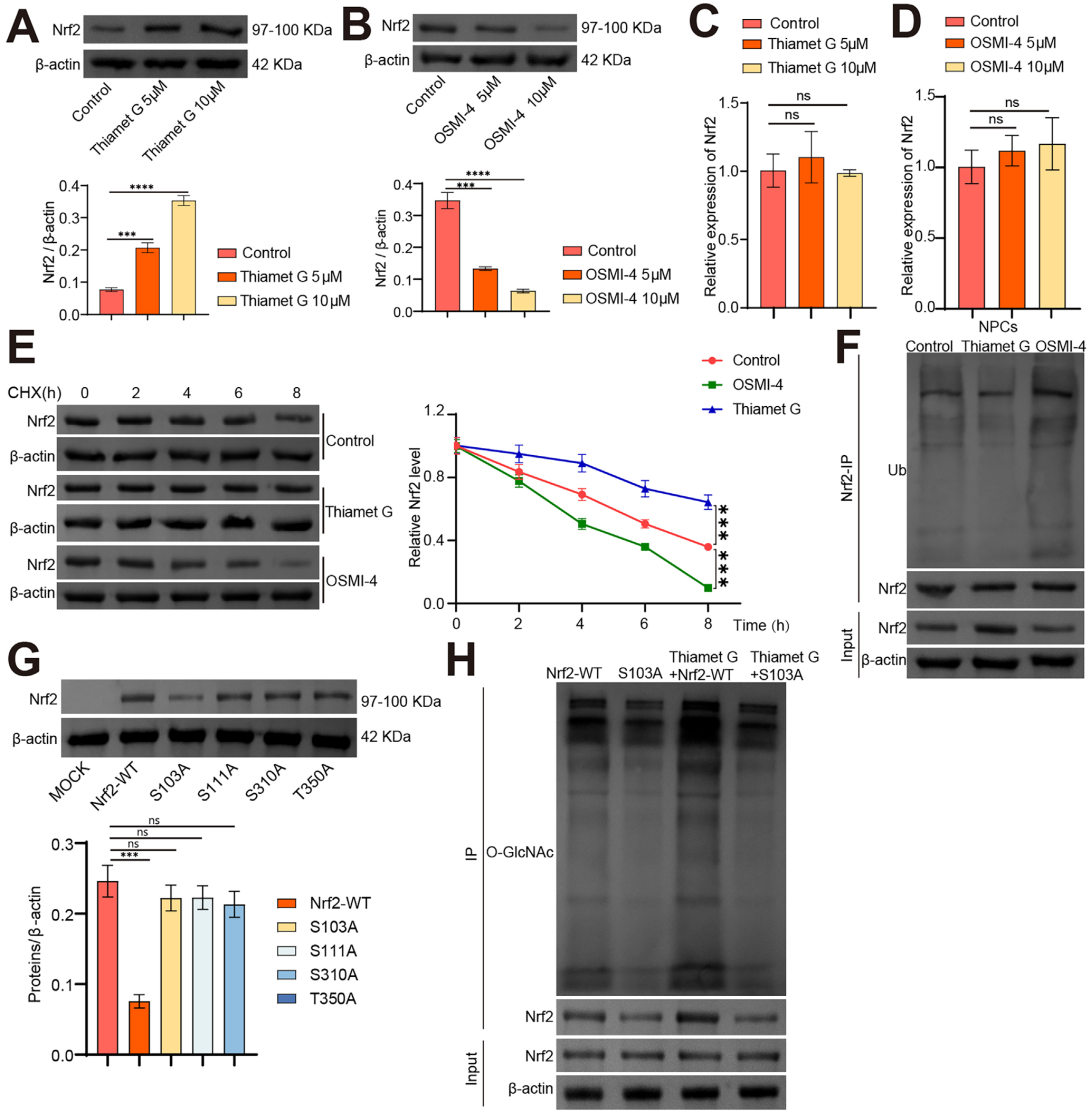
O‐GlcNAcylation stabilises Nrf2 expression through the ubiquitin‐proteasome pathway. (A, B) Nrf2 protein level was evaluated using WB. (C, D) Nrf2 mRNA level was assessed with qRT‐PCR. (E) Nrf2 protein stability was examined through the CHX chase test. (F) The ubiquitination level of Nrf2 was evaluated with IP. (G) NPCs were transfected with Flag‐labelled Nrf2‐WT, Nrf2‐S103A, Nrf2‐S111A, Nrf2‐S310A and Nrf2‐T350A respectively. The expression of Nrf2 was detected by WB 48 h later. (H) The O‐GlcNAc level of Nrf2 was detected by IP. *n* = 3. ***p < 0.001, ****p < 0.0001, ns indicates *p* > 0.05.

Prior research has shed light on the regulatory effect of O‐GlcNAcylation on Nrf2 [31]. We investigated if O‐GlcNAcylation could regulate Nrf2 expression in NPCs. WB results revealed that, compared to the control group, the Nrf2 protein levels tremendously increased after the O‐GlcNAc level was increased by adding OGA inhibitor Thiamet G (Figure 4A). After lowering O‐GlcNAc levels with the OGT inhibitor OSMI‐4, the protein levels of Nrf2 were significantly reduced (Figure 4B). However, qRT‐PCR results indicated that Thiamet G and OSMI‐4 did not affect Nrf2 mRNA levels (Figure 4C,D).

Next, NPCs were tested under Thiamet G or OSMI‐4 treatment and then exposed to CHX for 2, 4, 6, and 8 h. We observed that after CHX treatment, Nrf2 protein levels gradually declined as treatment time increased. Interestingly, Thiamet G slowed the rate of CHX‐induced Nrf2 protein degradation, while OSMI‐4 accelerated it (Figure 4E). Finally, after NPCs were pre‐incubated with Thiamet G or OSMI‐4, and subsequently treated with MG132, the ubiquitination level of Nrf2 was examined. The results of the IP experiment showed that, compared to the control group, Thiamet G reduced and OSMI‐4 raised the ubiquitination level of Nrf2 (Figure 4F).

Finally, based on the findings of Zhang et al. [32], which identified four potential O‐GlcNAcylation sites on Nrf2 (Ser103, Ser111, Ser310 and Thr350), we mutated these sites to assess their impact on the expression of Nrf2. The WB detection results showed that the S103A mutation was the sole alteration that led to a significant reduction in Nrf2 expression. This suggests that Ser103 might be the main site for Nrf2 O‐GlcNAcylation (Figure 4G). Next, we detected the O‐GlcNAc level of Nrf2 through IP. As shown in Figure 4H, compared with the Nrf2‐WT group, the O‐GlcNAcylation of Nrf2 in the Thiamet G + Nrf2‐WT group significantly increased, while a decrease was observed in the S103A group. Notably, the introduction of the S103 mutation resulted in no significant change in the O‐GlcNAcylation level of Nrf2 upon the addition of Thiamet G. These findings suggest that O‐GlcNAcylation stabilizes Nrf2 expression in NPCs via the ubiquitin‐proteasome pathway, with Ser103 identified as the primary site for Nrf2 O‐GlcNAcylation.

### 3.5 APS Mitigates NPCs Apoptosis and ECM Degradation Through Nrf2/HO‐1 Signalling

**FIGURE 5:**
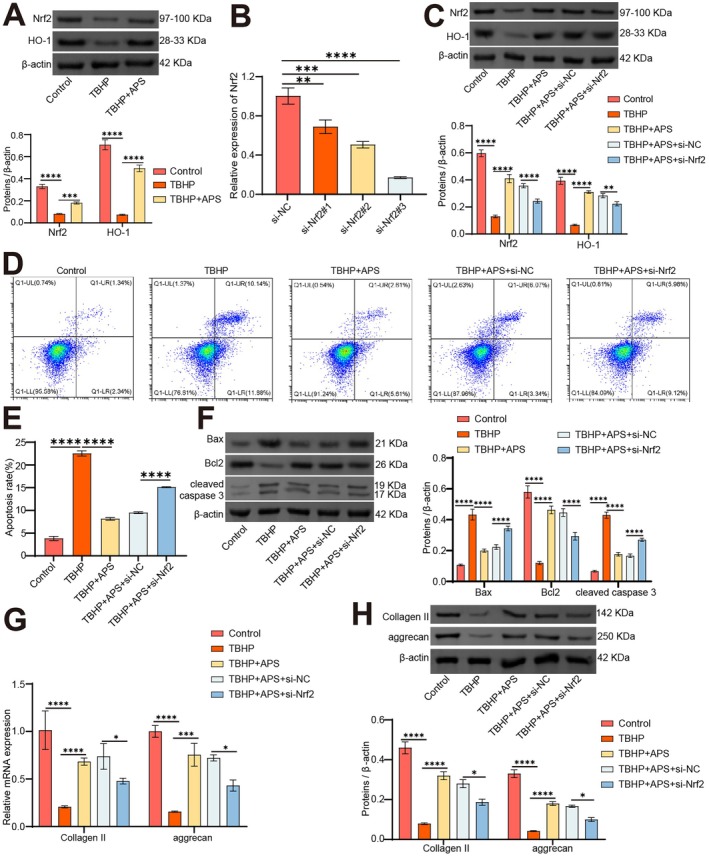
APS mitigates NPCs apoptosis and ECM degradation through Nrf2/HO‐1 signalling. NPCs were transfected with si‐Nrf2 and then APS and TBHP were added to investigate the role of Nrf2 in APS treatment of IVDD in vitro. (A) The expression levels of Nrf2 and HO‐1 were evaluated by WB. (B) Nrf2 expression levels were gauged using qRT‐PCR. (C) The expression levels of Nrf2 and HO‐1 were evaluated by WB. (D, E) NPCs apoptosis was assessed by Annexin V‐PI. (F) The expression levels of Bcl‐2, Bax, and cleaved caspase 3 were evaluated by WB. (G, H) The expression levels of Collagen II and aggrecan were examined by qRT‐PCR (G) and WB (H). *n* = 3. *p < 0.05, **p < 0.01, ***p < 0.001, ****p < 0.0001, ns indicates *p* > 0.05.

The above results have confirmed that APS can enhance O‐GlcNAcylation, and O‐GlcNAcylation can stabilize the expression of Nrf2. In this section, we aimed to investigate whether APS mitigates IVDD by modulating Nrf2/HO‐1 signaling at the cellular level. WB results showed that compared to the Control group, the protein levels of Nrf2 and HO‐1 were tremendously reduced in the TBHP group. However, compared to the TBHP group, the levels of Nrf2 and HO‐1 proteins were immensely elevated within the TBHP+APS group (Figure 5A).

We further analysed whether the downregulation of endogenous Nrf2 affects apoptosis and ECM degradation in the degraded NPCs. Initially, we screened and validated the knockdown efficiency of si‐Nrf2 using qRT‐PCR (Figure 5B). We then transfected the most efficient knockdown, si‐Nrf2, and treated NPCs with TBHP and APS. WB test results displayed immensely reduced Nrf2 and HO‐1 protein levels following Nrf2 knockdown, compared to the TBHP + APS + si‐NC group (Figure 5C).

Flow cytometry analysis revealed that levels of apoptosis obviously increased after si‐Nrf2 transfection compared to the TBHP + APS + si‐NC group (Figure 5D,E). The WB analysis showed elevated levels of Bax and cleaved‐caspase 3 protein, and reduced Bcl2 protein expression following Nrf2 knockdown, compared to the TBHP+APS + si‐NC group (Figure 5F). Furthermore, qRT‐PCR and WB results showed that, compared to the TBHP + APS + si‐NC group, the expression levels of Collagen II and aggrecan obviously decreased following Nrf2 knockdown (Figure 5G,H). These findings suggest that APS alleviates NPCs' apoptosis and ECM degradation by activating Nrf2/HO‐1 signalling.

### 3.6 APS Mitigates NPCs Apoptosis and ECM Degradation by Modulating O‐GlcNAcylation of Nrf2

**FIGURE 6:**
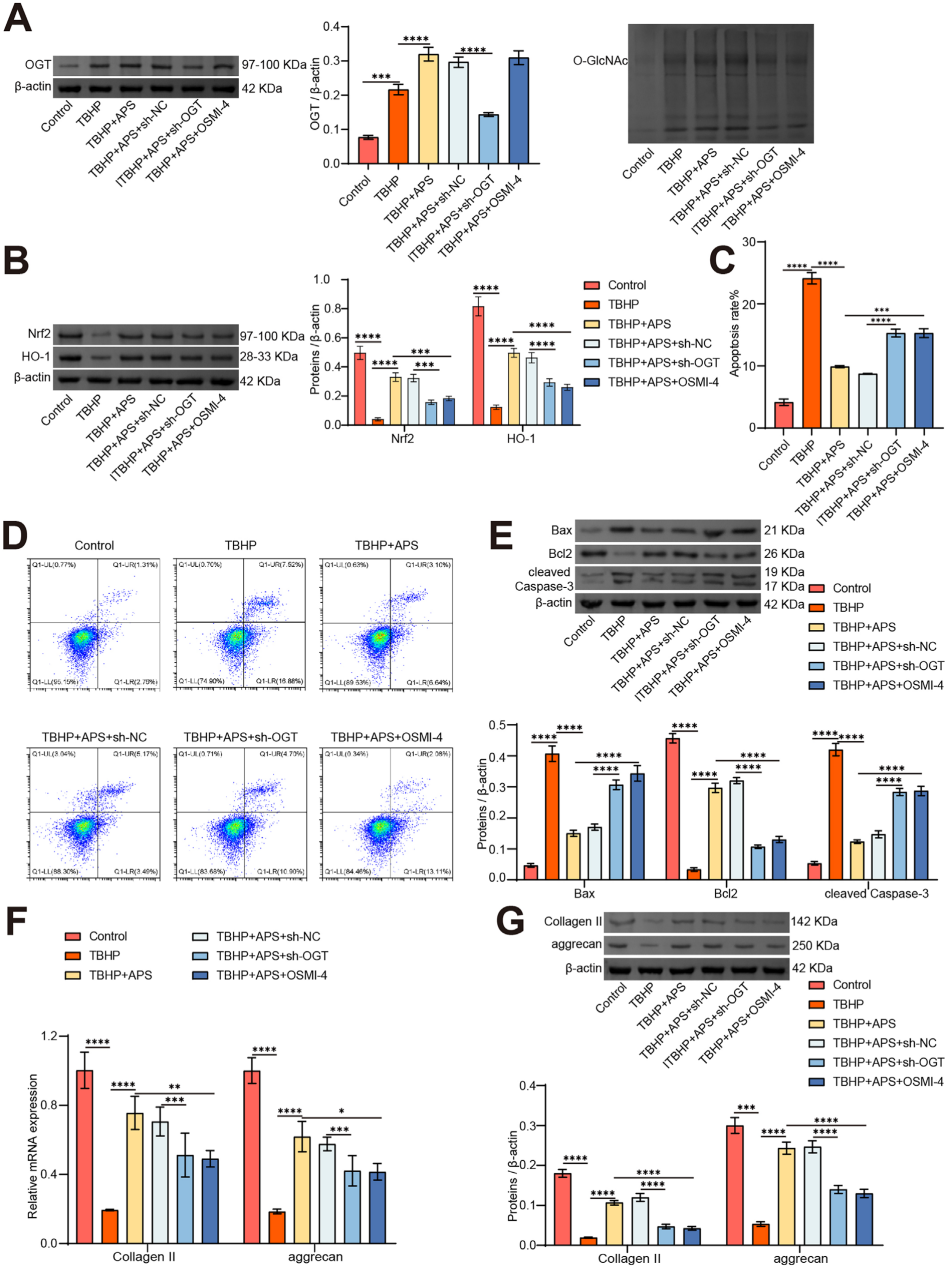
APS alleviates NPCs apoptosis and ECM degradation by mediating O‐GlcNAcylation of Nrf2. NPCs were transfected with sh‐OGT or OSMI‐4 and then treated with APS and TBHP to explore the role of O‐GlcNAcylation regulated Nrf2 expression in APS treatment of IVDD in vitro. (A) The OGT and O‐GlcNAc levels were detected by WB. (B) Nrf2 and HO‐1 expression levels were assessed using WB. (C, D) NPCs apoptosis statistics (C) and images (D). (E) Expression levels of Bcl‐2, Bax, and cleaved caspase 3 were evaluated by WB. (F, G) The expression levels of Collagen II and aggrecan were assessed by qRT‐PCR (F) and WB (G). *n* = 3. *p < 0.05, **p < 0.01, ***p < 0.001. ****p < 0.0001.

To explore the role of APS in activating the O‐GlcNAcylation‐mediated Nrf2/HO‐1 signalling axis in IVDD in vitro, we transfected NPCs with sh‐OGT or treated them with OSMI‐4, followed by APS and TBHP treatment. WB results revealed immensely lower levels of OGT and O‐GlcNAc in the TBHP + APS + sh‐OGT group than in the TBHP + APS + sh‐NC group. Compared to the TBHP + APS group, O‐GlcNAc levels vastly decreased in the TBHP + APS + OSMI‐4 group, while OGT expression showed no significant difference (Figure 6A). WB results indicated that Nrf2 and HO‐1 were noticeably lower in the TBHP + APS + sh‐OGT group and TBHP + APS + OSMI‐4 group compared to the TBHP + APS + sh‐NC group or APS group (Figure 6B).

Flow cytometry analysis demonstrated pronouncedly elevated apoptosis levels in the TBHP + APS + sh‐OGT and TBHP + APS + OSMI‐4 groups compared to the TBHP + APS + sh‐NC or TBHP + APS group (Figure 6C,D). Additionally, the changes in NPCs apoptosis were further confirmed based on WB results of Bcl‐2, Bax, and cleaved caspase 3 (Figure 6E). Lastly, qRT‐PCR and WB results indicated that the expression levels of Collagen II and aggrecan were pronouncedly lower in the TBHP + APS + sh‐OGT group and TBHP + APS + OSMI‐4 group compared to the TBHP + APS + sh‐NC group or TBHP + APS group (Figure 6F,G). These findings suggest that APS mitigates NPCs apoptosis and ECM degradation by enhancing OGT‐mediated O‐GlcNAcylation of Nrf2.

### 3.7 APS Mitigates IVDD Deterioration in Rats by Enhancing O‐GlcNAcylation of Nrf2

**FIGURE 7:**
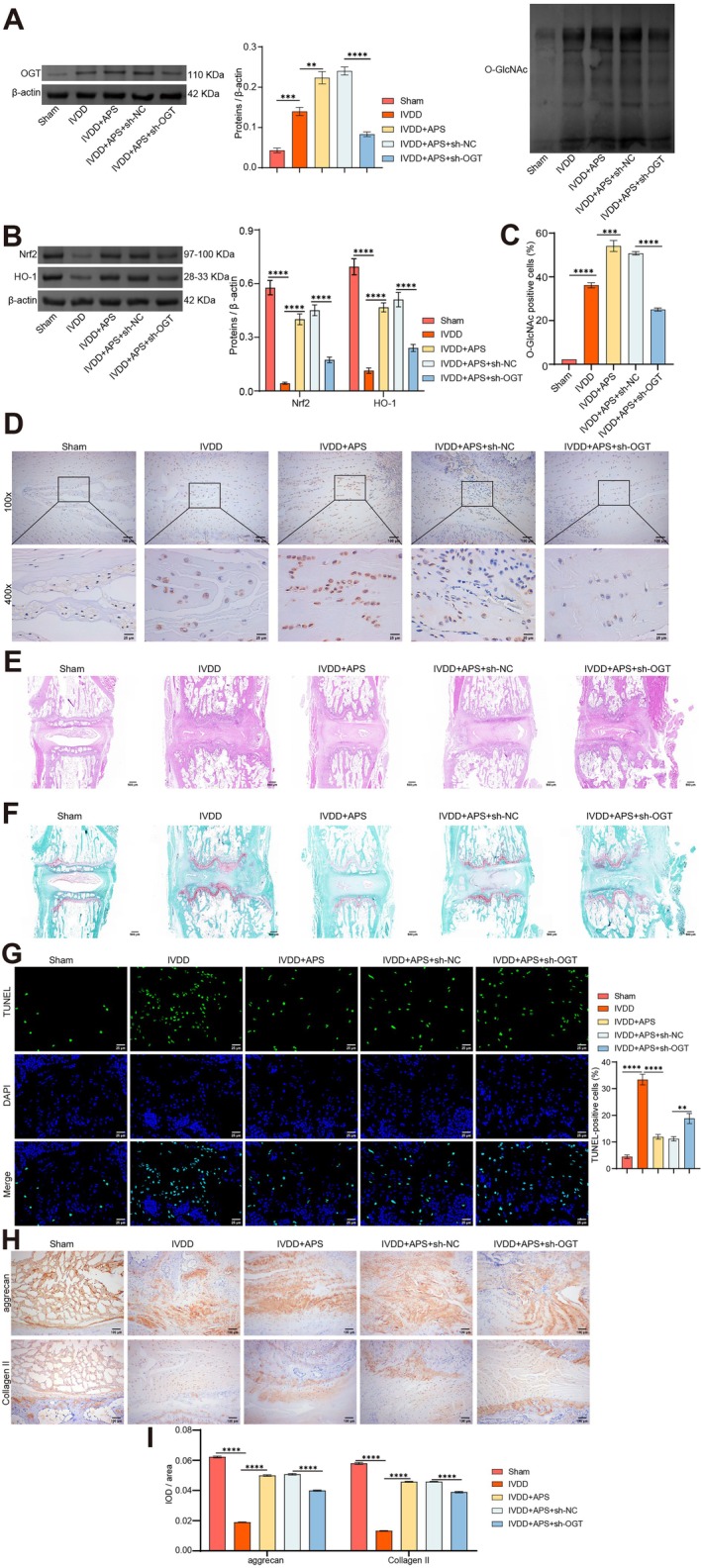
APS alleviates IVDD deterioration in rats by enhancing O‐GlcNAcylation of Nrf2. IVDD rats were treated with APS and OGT shRNA to explore the role of O‐GlcNAcylation regulated Nrf2 expression in the treatment of IVDD by APS. (A) WB was employed to determine OGT and O‐GlcNAc levels in intervertebral disc tissue. (B) Nrf2 and HO‐1 levels in intervertebral disc tissues were evaluated using WB. (C, D) IHC was used to detect O‐GlcNAc levels in intervertebral disc tissue. (E, F) Representative images of disc tissue stained with H&E and saffron O‐fast green. (G) TUNEL images of disc tissue and statistical analysis. (H, I) IHC images (H) and statistical analysis (I) of Collagen II and aggrecan. *n* = 5. **p < 0.01, ***p < 0.001. ****p < 0.0001.

Lastly, we analysed the effect of APS‐mediated O‐GlcNAcylation of Nrf2 on IVDD in vivo. Consistent with previous findings, WB results displayed elevated levels of OGT and O‐GlcNAc in IVDD rats following APS treatment. However, interfering with OGT expression pronouncedly counteracted the promotional effects of APS on OGT and O‐GlcNAc compared to the IVDD + APS + sh‐NC group (Figure 7A). WB findings showed that APS treatment notably elevated Nrf2 and HO‐1 expression in IVDD rats compared to the IVDD group. Notably, Nrf2 and HO‐1 expression levels reduced pronouncedly following OGT expression interference, compared to the IVDD + APS + sh‐NC group (Figure 7B). This change in O‐GlcNAc expression was further confirmed by IHC staining (Figure 7C,D).

Observations based on H&E and saffron O‐fast green staining, consistent with previous results, showed that APS treatment eased the deterioration of the intervertebral disc in IVDD rats, primarily characterised by a relatively intact NP and regular AF arrangement. However, compared to the IVDD + APS + sh‐NC group, the beneficial effects of APS on disc deterioration were partially negated following OGT expression interference (Figure 7E,F). TUNEL and IHC staining of disc samples revealed a pronounced reduction in the number of apoptosis‐positive cells and a marked increase in Collagen II and aggrecan expression following APS treatment, compared to the IVDD group. However, these effects were partially inhibited when OGT expression was interfered with, compared to the IVDD + APS + sh‐NC group (Figure 7G–I). These results suggest that APS mitigates IVDD deterioration in rats by enhancing O‐GlcNAcylation of Nrf2.

## 4 Discussion

IVDD is widely acknowledged to be a principal cause of LBP [33]. Functional changes in NPCs play a pivotal role in IVDD [29]. While existing evidence establishes a close connection between IVDD pathology and NPCs apoptosis and ECM metabolic disorders, current therapeutic approaches have not been successful in addressing these issues effectively [34]. This study marks the first report on the interventional role of APS in IVDD progression. Our findings suggest that APS inhibits NPCs apoptosis and ECM degradation by enhancing OGT‐mediated O‐GlcNAcylation of Nrf2, leading to an amelioration of IVDD deterioration in rats.

APS is known for its myriad biological activities, including antioxidative [35], anti‐inflammatory [36], anti‐aging [37], and immunomodulatory effects [38]. In view of the multifunctional nature of APS, we investigated its role in IVDD progression. Our study showed that APS administration to IVDD rats alleviated IVDD deterioration in a concentration‐dependent manner. Moreover, apoptosis of NPCs and ECM degradation have been identified as key players in the pathology of IVDD‐associated low back pain [39, 40]. TBHP has been reported to imitate the pathological conditions of IVDD in vitro [41, 42]. Hence, we examined the impact of APS on IVDD‐related NPC function in vitro. We found that APS restored NPC cell viability inhibited by TBHP stimulation in a concentration‐dependent manner. Further, we found that 0.8 mg/mL APS attenuates TBHP‐stimulated NPC cell apoptosis and ECM degradation. This was corroborated by the restoration of cell viability, augmented expression of Collagen II and aggrecan, and a reduction in apoptosis rates. This aligns with previous reports on the inhibitory effect of APS on apoptosis [43]. In conclusion, our study unveils, for the first time, the interventional role of APS in IVDD progression.

Recent studies have highlighted the protective effect of APS on cerebral ischemia–reperfusion injury through enhanced OGT‐induced O‐GlcNAcylation [30]. Based on these reports, we investigated APS's regulatory effects on O‐GlcNAcylation, OGT, and OGA within the context of IVDD. In line with previous findings [19], we confirmed that OGT and O‐GlcNAcylation levels were notably elevated in both intervertebral disc tissue of IVDD rats and TBHP‐treated NPCs, while OGA expression significantly decreased. Moreover, we observed that APS further increased OGT and O‐GlcNAcylation levels, and interestingly, APS treatment did not affect OGA expression. Cell function studies showed that APS greatly improved the apoptosis and ECM degradation of NPCs induced by TBHP stimulation. However, interference with OGT expression reversed these protective effects of APS on NPCs. This is in accordance with Luo et al.'s report, which outlined the inhibitory effects of increased O‐GlcNAcylation and OGT on NPCs' apoptosis and IVDD progression under nutrient deprivation [44]. These findings suggest APS improves IVDD via the promotion of O‐GlcNAcylation modification mediated by OGT.

Recent studies have reported functional crosstalk between O‐GlcNAcylation and NrF2‐mediated oxidative stress [45]. O‐GlcNAc signalling has been shown to mediate the positive regulation of Nrf2 and the negative regulation of apoptosis [31]. Costa RM et al. reported that the increase of O‐GlcNAc level induced by hyperglycemia can reduce Nrf2 activity and further promote oxidative stress damage [46]. Consistent with this study, we found that O‐GlcNAc levels increased and Nrf2 activity decreased in the context of IVDD‐induced pathological stress. Thiamet G is a potent and selective OGA inhibitor that increases O‐GlcNAc levels. OSMI‐4 is an OGT inhibitor that can reduce O‐GlcNAc levels [47]. Here, we found that Thiamet G or OSMI‐4 treatment promoted and inhibited Nrf2 protein expression in NPCs, respectively. Here, we found that Thiamet GH delayed the degradation rate of Nrf2 protein and inhibited Nrf2 ubiquitination, while OSMI‐4 accelerated the degradation rate of Nrf2 protein and promoted Nrf2 ubiquitination. These data suggest that OGT‐mediated O‐GlcNAcylation stabilises Nrf2 expression through the ubiquitin‐proteasome pathway. This finding is consistent with previous reports [32]. These studies suggest that APS may ameliorate IVDD by promoting O‐GlcNAcylation of Nrf2.

APS has previously been reported to induce the activation of the Nrf2 pathway, thereby providing a protective role in neurodegenerative diseases [48], vascular complications of diabetes [49], and cardiovascular and cerebrovascular diseases [50]. The activation of Nrf2/HO‐1 signalling can maintain REDOX homeostasis, protecting NPCs from apoptosis and ECM degradation [51, 52], thus improving IVDD [53, 54]. This study found that APS mitigates NPCs apoptosis and ECM degradation by activating the Nrf2/HO‐1 pathway. However, interfering with Nrf2 expression reversed the protective effect of APS on NPCs, which aligns with previous reports that knocking down Nrf2 promoted NPCs apoptosis [55]. Lu and colleagues discovered that transient upregulation of O‐GlcNAcylation can minimise pathological damage in IVDD mice [56]. In this research, we confirmed in vivo that APS activates the Nrf2/HO‐1 signalling axis through the upregulation of O‐GlcNAcylation and OGT, thereby mitigating IVDD deterioration in rats. Nevertheless, although an IVDD rat model was induced by puncture injury, it can't fully mimic the natural process of disc degeneration in humans. Moreover, while the current study has elucidated the mechanism by which APS improves IVDD through OGT/O‐GlcNAcylation‐mediated stabilisation of Nrf2, it is crucial to acknowledge that the pathogenesis of IVDD involves multiple signalling pathways, including NF‐κB [57], MAPK [58], and PI3K/Akt [59]. At present, this study has not thoroughly investigated whether APS exerts direct or indirect effects on other IVDD‐related signalling pathways via O‐GlcNAcylation. Therefore, future research will expand to more intricate regulatory networks to comprehensively elucidate the mechanisms underlying APS‐mediated improvements in IVDD.

In conclusion, our study revealed that APS can inhibit IVDD progression by reducing NPCs' apoptosis and ECM degradation within an IVDD context. The mechanism is related to the activation of the Nrf2/HO‐1 signalling axis by an OGT‐mediated increase of O‐GlcNAcylation. Notably, our study highlights for the first time the interventional role of APS in puncture injury‐induced IVDD rat models, potentially providing new preventative and treatment approaches for IVDD.

## Author Contributions

**Hao Tan:** data curation (lead), formal analysis (lead), funding acquisition (lead), methodology (lead), software (lead), validation (lead), writing – original draft (lead). **Cao Fang:** data curation (equal), formal analysis (equal), funding acquisition (equal), methodology (equal), project administration (equal), software (equal), validation (equal), writing – review and editing (equal). **Yiyun Tan:** data curation (equal), formal analysis (equal), investigation (equal), methodology (equal), visualization (equal), writing – review and editing (equal). **Zhi Wang:** data curation (equal), formal analysis (equal), resources (equal), software (equal), visualization (equal), writing – review and editing (equal). **Yun Zhou:** data curation (equal), formal analysis (equal), software (equal), visualization (equal), writing – review and editing (equal). **Xing Li:** conceptualization (lead), data curation (equal), formal analysis (equal), project administration (lead), software (equal), visualization (lead), writing – review and editing (lead).

## Ethics Statement

The study protocols were approved by Changsha Hospital of Traditional Chinese Medicine Animal Ethics Committee (No. 2023072011).

## Consent

The authors have nothing to report.

## Conflicts of Interest

The authors declare no conflicts of interest.

## Data Availability

All of the data is available from the corresponding author on reasonable request.
